# New isoforms and assembly of glutamine synthetase in the leaf of wheat (*Triticum aestivum* L.)

**DOI:** 10.1093/jxb/erv388

**Published:** 2015-08-24

**Authors:** Xiaochun Wang, Yihao Wei, Lanxin Shi, Xinming Ma, Steven M. Theg

**Affiliations:** ^1^Collaborative Innovation Center of Henan Grain Crops, Henan Agriculture University, Zhengzhou 450002, China; ^2^State Key Laboratory of Wheat and Maize Crop Science in China, Henan Agriculture University, Zhengzhou 450002, China; ^3^Department of Biochemistry, College of Life Science, Henan Agriculture University, Zhengzhou 450002, China; ^4^Department of Plant Biology, University of California-Davis, One Shields Avenue, Davis, CA 95616, USA

**Keywords:** Assembly, enzyme isoform, glutamine synthetase, nitrogen, protein modification, wheat.

## Abstract

Glutamine synthetase (GS; EC 6.3.1.2) plays a crucial role in the assimilation and re-assimilation of ammonia derived from a wide variety of metabolic processes during plant growth and development. Here, three developmentally regulated isoforms of GS holoenzyme in the leaf of wheat (*Triticum aestivum* L.) seedlings are described using native-PAGE with a transferase activity assay. The isoforms showed different mobilities in gels, with GS_II_>GS_III_>GS_I_. The cytosolic GS_I_ was composed of three subunits, GS1, GSr1, and GSr2, with the same molecular weight (39.2kDa), but different p*I* values. GS_I_ appeared at leaf emergence and was active throughout the leaf lifespan. GS_II_ and GS_III_, both located in the chloroplast, were each composed of a single 42.1kDa subunit with different p*I* values. GS_II_ was active mainly in green leaves, while GS_III_ showed brief but higher activity in green leaves grown under field conditions. LC-MS/MS experiments revealed that GS_II_ and GS_III_ have the same amino acid sequence, but GS_II_ has more modification sites. With a modified blue native electrophoresis (BNE) technique and in-gel catalytic activity analysis, only two GS isoforms were observed: one cytosolic and one chloroplastic. Mass calibrations on BNE gels showed that the cytosolic GS1 holoenzyme was ~490kDa and likely a dodecamer, and the chloroplastic GS2 holoenzyme was ~240kDa and likely a hexamer. Our experimental data suggest that the activity of GS isoforms in wheat is regulated by subcellular localization, assembly, and modification to achieve their roles during plant development.

## Highlight

Monomers of three developmentally regulated isoforms of glutamine synthetase holoenzyme in wheat cultivars were identified and their molecular weight was determined using modified blue native electrophoresis combined with an in-gel activity assay.

## Introduction

Glutamine synthetase (GS; EC 6.3.1.2) assimilates ammonium into glutamine, which is then used for the biosynthesis of all essential nitrogenous compounds (Miflin and Lea, 1977). All of the nitrogen within a plant, whether derived initially from nitrate, ammonium, N_2_ fixation, or catabolism of proteins, is channelled through reactions catalyzed by GS. Accordingly, GS plays a central role in nitrogen metabolism of vascular plants, and is a major checkpoint controlling plant growth and productivity (Brestic *et al.*, 2014; Habash *et al.*, 2007; Kichey *et al.*, 2006; Lothier *et al.*, 2011; Miflin and Habash, 2002; Simons *et al.*, 2014; Tabuchi *et al.*, 2005; Thomsen *et al.*, 2014).

In vascular plants, two isoforms of GS were initially resolved by chromatography (Mann *et al.*, 1979; McNally *et al.*, 1983; McParland *et al.*, 1976; O’Neal and Joy, 1973). Based on subcellular location, GS is classified as the cytosolic isoform (GS1) or the chloroplastic isoform (GS2). Electron microscopy analyses revealed that soybean (*Glycine max*) and common bean (*Phaseolus vulgaris*) GS enzymes are octamers (Llorca *et al.*, 2006; McParland *et al.*, 1976), whereas the crystallographic structures of GS in maize and *Medicago truncatula* are decamers (Torreira *et al.*, 2014; Unno *et al.*, 2006). GS2 is a single polypeptide (42–45kDa) encoded by one nuclear gene, whereas GS1 is composed of polypeptides with the same molecular weight (38–40kDa), but different p*I* values, and is encoded by three to five nuclear genes depending on the species. The GS isozymes have different metabolic roles, and their activities vary with plant development in different organs and cell types (Bernard *et al.*, 2008; Coque *et al.*, 2006; Finnemann and Schjoerring, 2000; Gallais *et al.*, 2006; Habash *et al.*, 2001; Kamachi *et al.*, 1991; Li *et al.*, 1993; Ohashi *et al.*, 2015; Orsel *et al.*, 2014; Tabuchi *et al.*, 2007). GS2 is the predominant isozyme in leaf mesophyll cells, where it assimilates ammonia originating from nitrate reduction and photorespiration (Kumagai *et al.*, 2011; Tobin and Yamaya, 2001). GS1 has multiple metabolic functions, involving primary ammonium assimilation in the roots, and catabolism ammonia re-assimilation for transport and distribution throughout the plant, and localizes to the vascular cells of various tissue of *Arabidopsis* (Guan *et al.*, 2015), wheat (*Triticum aestivum* L.) (Bernard *et al.*, 2008; Kichey *et al.*, 2005), rice (*Oryza sativa*) (Tabuchi *et al.*, 2005), tobacco (*Nicotiana tabacum*) (Brugiere *et al.*, 1999), and potato (*Solanum tuberosum*) (Pereira *et al.*, 1995). During leaf senescence, GS1 functions in the assimilation and recycling of the ammonia generated from catabolic processes (Avila-Ospina *et al.*, 2014; Bernard and Habash, 2009; Kamachi *et al.*, 1992). This role, confirmed by quantitative trait locus analysis, or gene mutation or knockout, is particularly important during grain development in cereals when nitrogen is remobilized to the reproductive sinks (Brestic *et al.*, 2014; Guan *et al.*, 2015; Martin *et al.*, 2006; Tabuchi *et al.*, 2005). To achieve these multiple non-overlapping roles, GS isozymes are regulated at the levels of transcription, translation, subcellular localization, assembly of subunits into the holoenzyme, post-translational modification of the enzyme, and protein turnover (Hirel *et al.*, 2001; Ishiyama *et al.*, 2004; Kamachi *et al.*, 1991; Li *et al.*, 1993; Lima *et al.*, 2006; Orsel *et al.*, 2014; Ortega *et al.*, 1999; Riedel *et al.*, 2001; Tabuchi *et al.*, 2007; Tobin and Yamaya, 2001). In wheat, seven genetic loci coding for three different forms of GS1 have been identified. *TaGS1a*, *TaGS1b*, and *TaGS1c* code for GS1;1, *TaGSr1* and *TaGSr2* code for GS1;2 (also called GSr), and *TaGSe1* and *TaGSe2* code for GS1;3 (also called GSe). Three alleles coding for GS2 (*TaGS2a*, *TaGS2b*, and *TaGS2c*) are known (Bernard *et al.*, 2008; Thomsen *et al.*, 2014). Here, three developmentally regulated GS holoenzymes in wheat are reported that can be separated by native-PAGE in plants.

There are several methods to analyze the oligomeric active state of a native protein, including gel filtration, analytical ultracentrifugation, electron microscopy, and X-ray crystallography. However, all of these methods require a substantial amount of protein and/or investment in expensive equipment. Blue native-PAGE [blue native electrophoresis (BNE)] and clear native-PAGE [clear native electrophoresis (CNE)] are performed with smaller amounts of protein and have been widely used to study membrane protein complexes (Filoni *et al.*, 2013; Strecker *et al.*, 2010; Wittig *et al.*, 2007; Wittig and Schagger, 2009). The application of these techniques to determine the native molecular weights and oligomeric states of the GS isoforms in wheat is reported here.

## Materials and methods

### Plant material and growth conditions

Wheat (*T. aestivum* L.) cvs Yumai 34, 49, and 50 were used for isolation of GS isoforms during the growth of first leaves in April in Zhengzhou, China. The other cultivars shown in Supplementary Fig. S1 (at *JXB* online) were grown similarly, but at different times of the year. The seeds were put in a disk covered with wet gauze at 25 °C until they germinated; they were then sown in individual pots (25cm upper diameter, 17cm lower diameter, and 25cm high) filled with vermiculite and grown outside under natural light/temperature. Each plant was sprayed with 300ml sterile water every day. The leaves were sampled three times from the onset of seedling emergence to the first leaf turning yellow, harvesting 0.5g per sample. Wheat cv. Yumai 49 was also grown under 10/14h light/dark periods at 23 °C in a growth chamber, with 800 μmol m^–2^ s^–1^ photon flux density at the top of the canopy during the light period, and watered with 200ml Hoagland solution (containing 1mM KH_2_PO_4,_ 5mM KNO_3_, 1mM MgSO_4_, 0.5mM CaSO_4_, 4mM Ca(NO_3_)_2_, 1mM Mg(NO_3_)_2_, 0.5mM CaCl_2_, 1 μM H_3_PO_3_, 1 μM CuSO_4_.5H_2_O, 1 μM MnCl_2_.4H_2_O, 1 μM Na_2_MoO_4_, and 1 μM ZnSO_4_.7H_2_O) twice a week to keep the soil moist and supply sufficient nutrients. Fully expanded green leaves were collected for the preparation of intact chloroplasts and enzyme analysis when the wheat seedlings had four or five leaves.

### Preparation of leaf extract

Sample (0.5g each) were ground into powder in a chilled mortar with liquid N_2_ and mixed with 1.5ml Extraction Buffer (100mM Tris, 1mM EDTA, 1mM MgCl_2_, and 10mM β-mercaptoethanol, pH 7.6). The extract was centrifuged at 13 000 *g* at 4 °C for 30min. The supernatant was prepared for native gel analysis.

### Isolation of chloroplasts

Intact chloroplasts were isolated essentially as described by Theg *et al.* (1989). The washed chloroplast pellet after the Percoll step was resuspended in 350 μl Extraction Buffer and incubated on ice for 10min to break the organelles. The resuspension was used for immunoblotting or centrifuged at 13 000 *g* for 10min and the supernatant used for enzyme assays immediately.

### In-gel detection of GS activity and molecular weight

Four gel systems (running at 4 °C) were used to separate the proteins and detect GS activities in the gel. First, a discontinuous native-PAGE system was used according to Robert and Wong (1986). The native gel system employed a 1.5 mm×170 mm×100mm gel, the analyzing gel was composed of 5% polyacrylamide (pH 8.7), and the stacking gel was 3% polyacrylamide (pH 6.7). Samples were normalized to 30 μl (~60 μg protein) from 0.5g FW leaves in each lane, and electrophoresis was carried out at 80V for the stacking gel and 120V for the resolving gel at 4 °C. Second, the BNE system was used according to Wittig *et al.* (2006, 2007) with the following modifications. The sample gel contained 3.5% polyacrylamide and the gradient resolving gel contained 4–13% polyacrylamide; the gel was 1.5 mm×170 mm×100mm, the sample buffer was Extraction Buffer, and before loading sample in the gel, 100 μl sample was mixed with 10 μl 50% glycerol. The current was limited to 15 mA during electrophoresis. The gel was run with cathode buffer A (0.02% Coomassie Blue G250, 50mM Tricine, and 5mM imidazole, pH 7.0) until the blue dye front was up to half of the gel length; cathode buffer A was then removed and the gel was run with cathode buffer B (0.002% Coomassie Blue G250, 50mM Tricine, and 5mM imidazole, pH 7.0) until the blue dye front moved out of the gel. Third, the BNE protocol was modified as follows. The gradient gel was prepared as the second gel system. After the gel ran for 1h with cathode buffer A, cathode buffer A was removed and the gel was run with cathode buffer C (50mM Tricine and 5mM imidazole, pH 7.0) until the blue dye front moved out of the gel. Fourth, for CNE, the gradient gel was prepared in the second gel system. Each well received 50 μl cathode buffer A before the sample was loaded. The gel was run only with cathode buffer C (50mM Tricine and 5mM imidazole, pH 7.0) until the blue dye front moved out of the gel.

After electrophoresis, GS activity was detected in-gel by the conversion of l-glutamine to γ-glutamyl hydroxamate (Barratt, 1980). The gel was immersed in 100ml reaction buffer (100mM Tricine, 1.3mM EDTA, 20mM sodium arsenate, 20mM MgSO_4_, 0.5mM ADP, 25mM hydroxylamine, and 50mM l-glutamate, pH 7.4) and incubated at 37 °C for 45min with slow shaking, after which the reaction buffer was removed. The reaction was terminated by adding 50 μl stop solution (370mM FeCl_3_, 200mM trichloroacetic acid, and 700mM HCl) for ~3min until GS activity appeared as a brownish band in the yellow background. The gel was washed twice with cool distilled H_2_O and scanned immediately. The GS bands were marked with a blade and then the gel was stained with Coomassie Blue R250. The molecular mass of the GS isoforms was calculated by comparison with molecular weight standards (Life Technologies) using Quantity One software.

### GS recovery and GS subunit identification

After the GS activity was detected in the gel, the band of interest was excised with a scalpel, rinsed with 0.5mM EDTA, pH 7.6, and ground in a chilled mortar with this same solution. The homogenate was centrifuged at 12 000 *g* at 4 °C for 20min and then the extraction was mixed with an equal volume of 0.1M Tris-buffered phenol (pH 8.0). After being centrifuged (12 000 *g*) at 4 °C for 20min, the protein in the phenol phase was precipitated with 4 vols 0.1M ammonium acetate in methanol overnight at –20 °C. The proteins recovered by centrifugation were washed once with 1ml cold methanol and twice with 1ml cold acetone, and then resolved in SDS sample buffer for analysis. A discontinuous SDS-PAGE system was implemented according to Laemmli (1970), with a 12.5% polyacrylamide analyzing gel and a 6% polyacrylamide stacking gel, and electrophoresis was performed at room temperature. Proteins were transferred to polyvinylidene difluoride membranes for blot analysis. GS polypeptides were detected using polyclonal antisera (generously provided by Bertrand Hirel) raised against GS2 of tobacco (Bernard *et al.*, 2008)

### Protein extraction for two-dimensional immunoblots

Protein was extracted using a modification of the phenol-based method (Finnemann and Schjoerring, 2000). Wheat leaves were homogenized in an ice-cold mortar and pestle in SDS sample buffer (0.1M Tris-Cl, 2% SDS, 5% 2-mercaptoethanol, and 30% sucrose, pH 8.0) and then mixed with the same volume of Tris-buffered phenol (pH 8.0). The homogenate was centrifuged at 10 000 *g* for 5min at 4 °C. Protein in the upper phenol phase was precipitated with 5 vols 0.1M ammonium acetate in methanol for 30min at –20 °C. The protein recovered by centrifugation was washed twice with cold 80% acetone and then dissolved in SDS sample buffer or rehydration buffer (8M urea, 4% CHAPS, 2% IPG buffer, pH 4–7, and 20mM DTT). Protein was quantified by the Bio-Rad protein assay with BSA as standard. For two-dimensional gel electrophoresis, wheat leaf proteins (600 μg) were loaded on to pH 4–7 Immobiline Drystrips (7cm; Amersham) by passive rehydration overnight at room temperature. The rehydrated strips were resolved in a Multiphor II apparatus (Pharmacia Biotech) by isoelectric focusing for 8000 Vh at 10 °C. The resolved strips were consecutively equilibrated in DTT solution (50mM Tris, 6M urea, 30% glycerol, 2% SDS, 0.002% bromophenol blue, and 1% DTT, pH 8.8) and iodoacetamide solution (50mM Tris, 6M urea, 30% glycerol, 2% SDS, 0.002% bromophenol blue, and 2.5% iodoacetamide, pH 8.8) for 15min, and the secondary SDS–PAGE was run with 12.5% gels. After electrophoresis, immunodetection was performed as described above.

### Protein identification by LC-MS/MS

After GS activity was detected in a native-PAGE gel, each band of interest was excised with a scalpel and washed with 75% ethanol. The samples were sent to the Genome Center at the University of California-Davis for identification of GS proteins and modifications by LC-MS/MS, and analyzed with Scaffold 4.0 software.

## Results

### Three isoforms of GS are active during wheat leaf development

**Fig. 1.**
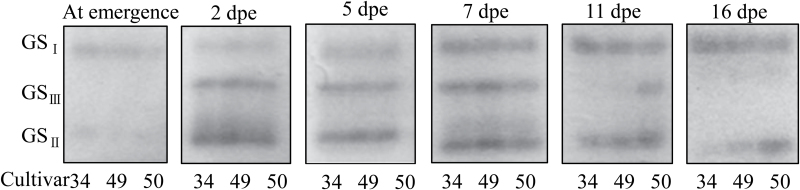
GS isoforms as a function of leaf development in wheat. GS isoforms were monitored using native-PAGE (5%) in the first leaf in seedlings of wheat cvs Yumai 34, 49. and 50. Samples were taken at the indicated times; dpe, days post-emergence.

To elucidate the role of GS isoforms during wheat development, leaf extracts from three cultivars of wheat seedlings at different developmental stages were separated by native-PAGE and GS isoforms were detected using transferase activity staining. Three isoforms of GS holoenzyme were identified in the wheat leaf. GS_I_, GS_II_, and GS_III_ emerged sequentially with the development of the first leaf (Fig. 1). Conversely, GS_III_, GS_II_, and GS_I_ disappeared in turn with leaf senescence (Fig. 1). GS_II_ had the highest mobility in native-PAGE, followed by GS_III_ and then GS_I_. GS_I_ was present during the seed germination stage and increased progressively in activity until leaf senescence. GS_II_ appeared with leaf expansion and maintained the highest activity in green leaves, but disappeared when the leaf turned yellow. GS_III_ had a shorter period of activity, albeit with higher activity, in the growing green leaf, i.e. from the stage of fast leaf expansion (Fig. 1, panels 2dpe and 5 dpe) to the full-length size (Fig. 1, panel 7dpe). It was deduced that GS_I_ was likely cytosolic (GS1) because it was present from the onset of germination until leaf senescence. GS_II_ was considered likely to be chloroplastic (GS2) because it was the dominant GS in green leaves. GS_III_ has not been described before.

### Subunit composition and subcellular localization of GS isoforms

**Fig. 2.**
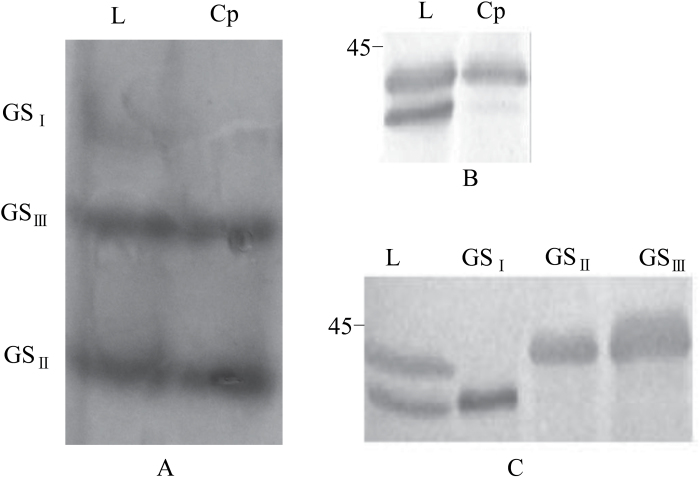
Electrophoretic separation and detection of cytosolic and chloroplastic GS, and GS-related subunits. (A) In-gel detection of GS activity. Protein extracts were prepared from leaves (L) and purified chloroplasts (Cp), separated by 5% native-PAGE, and GS isoforms were detected based on GS activity in the gel. (B) Detection of chloroplastic GS-related subunits. Proteins from the leaf or isolated chloroplasts were separated by 12.5% SDS-PAGE, and probed with antibodies against tobacco GS2. (C) Subunit composition of GS isozymes of wheat leaf. Protein extracts were separated using 5% native PAGE. GS isozyme bands were recovered by chemical extraction, separated by 12.5% SDS-PAGE, and probed with anti-tobacco GS2.

To characterize the GS isoforms further, chloroplasts were isolated and GS activity therein detected using native-PAGE. Both GS_II_ and GS_III_ were found in chloroplasts (Fig. 2A) and immunoblots revealed them to be derived from only one GS polypeptide of 43.6kDa (Fig. 2B). Based on these findings, they are considered to be chloroplastic GS2-type isoforms. By contrast, GS_I_ was not found in chloroplasts and so might be cytoplasmic. To confirm the identities of the GS isoforms, leaf proteins were separated by native-PAGE, and the bands displaying GS activity were recovered from the gel by chemical extraction, separated by SDS-PAGE, and detected by immunoblot. The band giving GS_I_ activity was composed of a 39kDa subunit (Fig. 2C), consistent with its identification as cytosolic GS1. The bands showing GS_II_ and GS_III_ activity were found to be composed of a single 43.6kDa subunit (Fig. 2C). This suggests that differential modifications of GS2-related subunits might confer different mobility to the holoenzymes (GS_II_ and GS_III_). Although the subunit of GS2 was bigger than that of GS1 (43.6 versus 39kDa), GS_II_ and GS_III_ ran faster than GS_I_ in the native-PAGE system, suggesting they may have different oligomeric states.

### Identification of GS protein sequences and modifications

To determine unambiguously the proteins corresponding to GS_I_, GS_II_, and GS_III_, bands containing these activities were excised from native gels and analyzed by LC-MS/MS (Supplementary Table S1 at *JXB* online). Protein identification revealed that the GS_I_ band contained fragments of three previously described cytosolic GS isoforms, GS1, GSr1, and GSr2 (equivalent to GS1;1 and two forms of GS1;2), although no GSe (equivalent to GS1;3) was detected. The complete sequences of GS_II_ and GS_III_ were obtained in the LC-MS/MS experiment; they were identical with a theoretical molecular weight of 42.1kDa, identical to GS2a, GS2b, and GS2c. The LC-MS/MS data (parent error <5 ppm) indicated that GS_II_ had many more modifications than did GS_III_, including acetylation, oxidation, dioxidation, and deamidation. In comparison, GS_III_ had fewer sites of oxidation, one site of acetylation, and more sites of deamidation (Table 1).

**Table 1.** Protein modifications detected in GS_II_ and GS_III_

**Isoform**	**Oxidation**	**Acetyl**	**Deamidation**	**Dioxidation**
**GS**_**II**_				
Sites	7	3	7	6
Amino acid	M W Q N	A L G	Q N	W M
**GS**_**III**_				
Sites	4	1	9	5
Amino acid	M W Q N	L	W Q N	W M

**Fig. 3.**
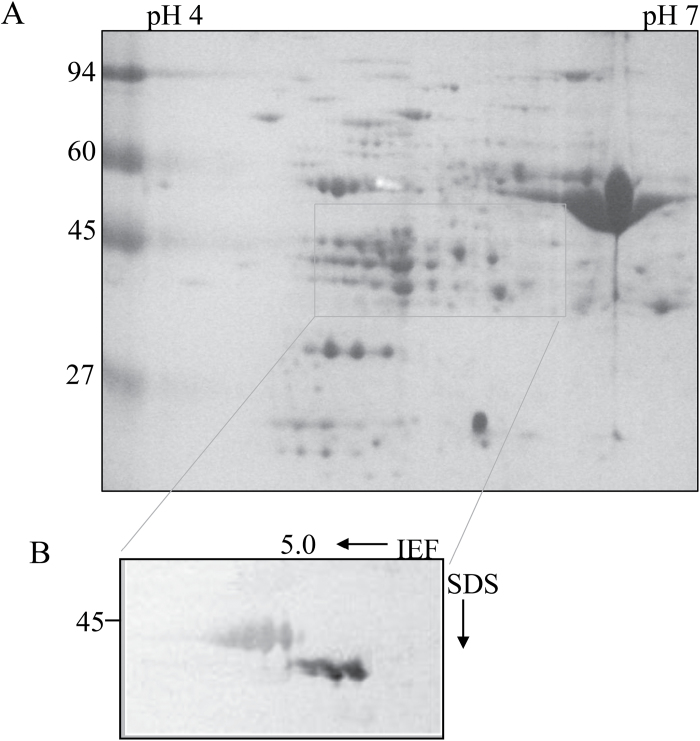
Two-dimensional analysis of GS isozymes in wheat leaves. (A) Two-dimensional gel of proteins extracted from the wheat leaf; stained with Coomassie Blue R250. The rectangle shows the region putatively containing GS spots. (B) Two-dimensional immunoblots of GS subunits in wheat leaf. IEF, isoelectric focussing. Leaf proteins (50 μg) were separated by two-dimensional electrophoresis, and the region thought to contain GS (larger than the rectangle) was electroblotted and probed with antibodies against tobacco GS2.

Two-dimensional separation of leaf proteins and subsequent immunoblotting revealed two groups of GS polypeptides with distinct p*I* values. In one, three 39kDa GS1-related polypeptides were detected with p*I* values of 5.08, 5.13, and 5.21 (Fig. 3). In the other, three 43.6kDa GS2-related polypeptides were detected with p*I* values of 4.8, 4.94, and 5.05. These combined data suggest that the GS_II_ and GS_III_ activity bands seen in native-PAGE were each composed of GS2-related proteins with different p*I* values due to different modifications.

### Oligomers of GS isoenzymes

**Fig. 4.**
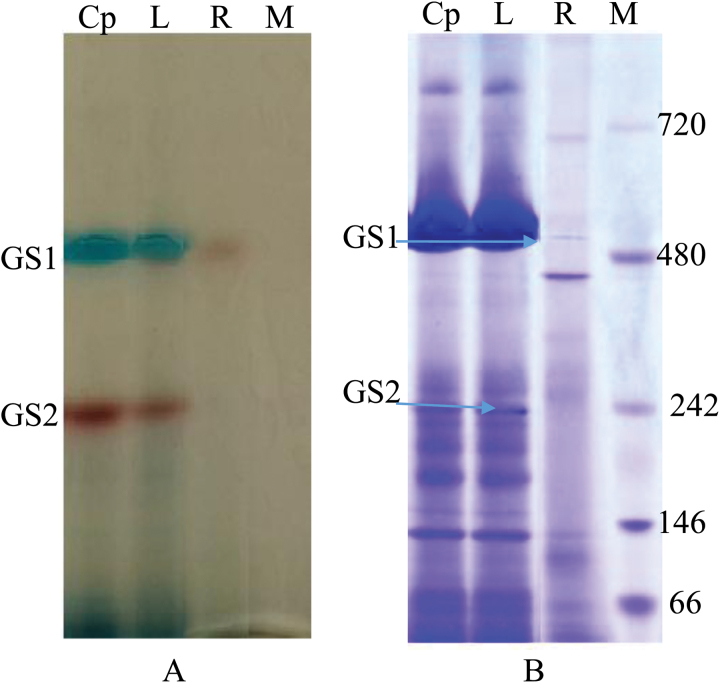
Identification of the native molecular weight of GS isoforms using BNE. (A) Soluble proteins (~100 μg) from wheat seedling chloroplasts (Cp), leafs (L), and roots (R) were separated by BNE on a 4–13% polyacrylamide gradient gel, and GS isoforms were detected with a transferase activity assay. (B) The GS bands were marked and the gel stained with Coomassie Blue R250. M, high-molecular-weight markers (mass in kDa given to the right of the gel). (This figure is available in colour at *JXB* online.)

The authors next turned their attention to elucidation of the oligomeric state of the wheat GS isoforms. In BNE, protein complexes are separated according to size in acrylamide gradient gels and their sizes can be calibrated with standards. During the initial BNE experiments, the presence of the Coomassie Brilliant Blue G250 (referred to hereafter as G250) interfered with the activity stain for GS1 (Supplementary Fig. S2 at *JXB* online). To overcome this the BNE protocol was modified to include a 1h separation of proteins in the presence of G250 and then an additional 3h separation in which the cathode buffer was replaced with one lacking the dye. This allowed a separation of the protein complexes by molecular weight and subsequent detection by the transferase activity stain. Fig. 4 shows the results of such an analysis and reveals that the GS1 holoenzyme has a molecular weight of ~490kDa. Given the molecular weight of the monomer (39kDa), this indicates that the GS1 holoenzyme is likely a dodecamer. Interestingly, GS2, containing both GS_II_ and GS_III_ activities, ran as a single band with a molecular weight of 240kDa, suggesting that the holoenzyme is most likely a hexamer. These results additionally suggest that while GS1 and GS2 have distinct migrations in native-PAGE gels, in part due to different oligomeric states of their respective holoenzymes, the different mobilities of GS_II_ and GS_III_, both GS2 isoforms, in the same native gel system must be due in part to their different modifications as described in Table 1.

## Discussion

In vascular plants, only two isoforms of the GS holoenzyme have been resolved by standard chromatography (Mann *et al.*, 1979; McNally *et al.*, 1983; McParland *et al.*, 1976; O’Neal and Joy, 1973) and by native gel electrophoresis (Nagy *et al.*, 2013; Péscsvéradi *et al.*, 2009). Here, three isoforms of GS were separated using native gels in wheat seedlings (Figs 1 and 2); the third isoform, GS_III_, has not been reported before. The fact that all three isoforms were observed in >20 cultivars (Supplementary Fig. S1 at *JXB* online) confirms they are generally present in wheat seedlings. In general, GS_III_ is readily observed in plants grown in the field, but is more difficult to detect in those grown in growth chambers. This might be ascribed to the lower light intensity of the growth chamber environment (<1000 μmol m^–2^ s^–1^ photon flux density), as opposed to sunlight which can provide ~2000 μmol m^–2^ s^–1^ photon flux density of photosynthetically active radiation in the field. The appearance of GS_III_ is also regulated with the leaf development independent of whether the wheat seedlings were grown without (Fig. 1) or with (Supplementary Fig. S3 at *JXB* online) nitrogen. These two factors may be responsible for it not having been identified in previous studies (Nagy *et al.*, 2013; Péscsvéradi *et al.*, 2009). For instance, GS_III_ was not found in green leaves of 14-d-old seedlings growing in chambers, whereas it was abundant in green leaves of 21-d-old seedlings from the same chamber (Fig. 2A).

The early estimates for the molecular weight of GS oligomers came from direct measurements (gel filtration, sedimentation equilibrium) with purified protein (Mann *et al.*, 1979; McParland *et al.*, 1976). It is difficult to obtain sufficient quantities of purified protein from plants for assembly and structure studies, and, consequently, these previous GS structural studies used proteins heterologously expressed in *Escherichia coli* (Llorca *et al.*, 2006; O’Neal and Joy, 1973; Seabra *et al.*, 2009; Torreira *et al.*, 2014; Unno *et al.*, 2006). BNE techniques provide an independent method for separating protein complexes with high resolution and from which their molecular weights can be determined. When soluble proteins from leaves or isolated chloroplasts were separated by BNE and GS isoform activities were detected in the gel, only one GS isoform (corresponding to chloroplastic GS2) was observed (Supplementary Fig. S2 at *JXB* online). This is likely because cytosolic GS1 activity appears to be sensitive to G250 and because Rubisco runs in the same part of the gel, overwhelming the transferase signal. When leaf or chloroplast soluble proteins were separated using the BNE procedure, but omitting G250 from the cathode buffer, two GS isoforms were detected in leaf extracts (GS1 and GS2) and one in the chloroplast (GS2), although chloroplastic GS in leaves and chloroplasts displayed slightly different mobility (Supplementary Fig. S4 at *JXB* online). Finally, the BNE protocol was modified, first running the gel with cathode buffer containing 0.02% G250 for 1h to ensure that all proteins carried same net charge and then changing to cathode buffer without G250 to reduce the influence of G250 on GS activity. Although cytosolic GS activity was weak, it was detectable. Furthermore, chloroplastic GS in leaf extracts and chloroplast extracts had the same mobility in the gel.

Based on this modified BNE procedure, the cytosolic GS, with the activity in GS_I_, was likely to be a dodecamer, the same as GSr1 in soybean nodules analyzed by analytical ultracentrifugation and native-PAGE (Masalkar and Roberts, 2015) and GS in prokaryotes (Eisenberg *et al.*, 2000). Our data suggest that wheat chloroplastic GS, with activities in GS_II_ and GS_III_, was a hexamer, which differs markedly from other plants. For example, GSs from soybean (McParland *et al.*, 1976) and common bean (Llorca *et al.*, 2006) are octamers as determined by electron microscopy; GSs from maize and *M. truncatula* and GS1β from soybean are decamers (Masalkar and Roberts, 2015; Torreira *et al.*, 2014; Unno *et al.*, 2006) as determined by X-ray crystallography. Separated by CNE and detected by in-gel GS activity assay, GS from spinach stroma is a decamer (Kimata-Ariga and Hase, 2014). Confidence in the methodology for oligomeric state determination is strengthened by Supplementary Fig. S5 (at *JXB* online) in which maize GS can be seen running as a decamer. Nonetheless, the authors recognize that the oligomeric state reported here should be further evaluated by additional techniques, and future plans call for expression of recombinant *TaGS1* and *TaGS2* and analysis by X-ray crystallography. The authors are also working to compare GS proteins in *M. truncatula*, soybean, *Arabidopsis*, spinach, and common bean with those in wheat using the modified BNE system.

The different p*I* values for the wheat GS proteins have different origins. GS_I_, cytosolic GS, is encoded by a multigene family, *GS1* and *GSr*, and the p*I* values detected here are close to those predicted by analysis of the respective gene sequences (Bernard *et al.*, 2008). However, no GSe was identified by MS analysis, perhaps because its expression was too low to be detectable in leaves and roots during the wheat seedling stage (Bernard *et al.*, 2008). In contrast, chloroplastic GS is encoded by three alleles (*TaGS2a*, *TaGS2b*, and *TaGS2c*), and the different p*I* values must arise from different post-translational modifications. Lima (2006) reported that phosphorylated GS2 of *M. truncatula* interacts with 14-3-3 proteins, which leads to selective proteolysis and thus inactivation of the plastid isoform. In *E. coli*, GS is reported to be inactivated by adenlylation (Liaw *et al.*, 1993), and oxidation of soybean root GS has been reported to lead to its inactivation and increased susceptibility to degradation (Ortega *et al.*, 1999). No evidence for phosphorylation of GS2 was found here, but numerous other modifications were detected, and they were different for GS_II_ and GS_III_ (Supplementary Fig. S6 at *JXB* online). For instance, GS_II_ had three acetylation sites in its N-terminal region, whereas GS_III_ had one such site. GS_II_ had seven sites of oxidation, while GS_III_ had four, even though GS_II_ activity was higher than that of GS_III_ in all but the most active stages of leaf development (Fig. 1). Whether the various modifications regulate GS2 enzyme activity or stability remains to be established.

Recently, an analysis of GS in *Arabidopsis* was presented in which 11 different GS1 isoforms were detected in a 7% resolving gel using a phosphate release assay and no GS2 was observed (only this group detected GS activity using this method) (Dragicevic *et al.*, 2014). This is clearly different from the situation described herein for wheat and emphasizes the potential diversity of GS assembly configurations in different plant species.

When GS isoenzymes were originally discovered, their putative functions were deduced from their pattern of expression in different tissues during plant development and further confirmed by genetic methods (Bao *et al.*, 2014; Gadaleta *et al.*, 2011; Gadaleta *et al.*, 2014; Guo *et al.*, 2013; Habash *et al.*, 2007; Martin *et al.*, 2006). GS1, vascular-localized cytosolic GS, is proposed to be involved in the re-assimilation of ammonium released during leaf senescence and in transporting ammonium from source organs to sink organs, e.g. from fully expanded leaves to new leaves (Bernard *et al.*, 2008; Kichey *et al.*, 2006; Kichey *et al.*, 2007). GS2, however, was found in both mitochondria and chloroplasts in *Arabidopsis* (Taira *et al.*, 2004), suggesting that this isoform is active in re-assimilation of the large pool of ammonia released by photorespiration. It is noteworthy that neither GS activity nor GS subunits in mitochondria purified from wheat leaves were detected in the present report (data not shown). This, along with the detection of GS_III_ primarily in leaves grown under relatively high light intensity, would be consistent with a function of chloroplastic GS2 in original nitrogen assimilation, especially under conditions of abundant energy availability that would promote the conversion of nitrate to ammonium in the plastid. Although the physiological role of the newly described GS_III_ remains to be elucidated, findings presented here suggest that there is a complex and flexible regulation for GS isoforms in wheat that is coupled to nitrogen utilization and plant growth.

## Supplementary data

Supplementary data are available at *JXB* online.

Figure S1. GS isoforms in the leaf of different wheat cultivars.

Figure S2. GS isoforms in wheat chloroplasts, leaves, and roots.

Figure S3. GS isoforms as a function of leaf development in wheat.

Figure S4. GS isoforms in wheat chloroplast, leaf, and roots.

Figure S5. GS isoforms in wheat leaf and roots, and maize leaf and roots.

Figure S6. Amino acid modifications sites in GSII and GSIII.

Table S1. Identification of the composition of GS_I_, GS_II_, and GS_III_ by MS analysis.

Supplementary Data

## Abbreviations:

BNE: blue native electrophoresis
CNE: clear native electrophoresis
GS: glutamine synthetase.
