# Supplementary material for: New isoforms and assembly of glutamine synthetase in the leaf of wheat (Triticum aestivum L.)
Source: J Exp Bot. 2015 Aug 24;66(21):6827–34. doi: 10.1093/jxb/erv388 (PMC4623691; doi:10.1093/jxb/erv388)
Supplement: Supplementary Data [file supp_66_21_6827__index.html]

New isoforms and assembly of glutamine synthetase in the leaf of wheat (Triticum aestivum L.) — Supplementary Data 

# New isoforms and assembly of glutamine synthetase in the leaf of wheat (*Triticum aestivum* L.)

## Supplementary Data

Data files

- Supplementary Data - Supplementary Data
