# Supplementary material for: New isoforms and assembly of glutamine synthetase in the leaf of wheat (Triticum aestivum L.)
Source: J Exp Bot. 2015 Aug 24;66(21):6827–34. doi: 10.1093/jxb/erv388 (PMC4623691; doi:10.1093/jxb/erv388)
Supplement: Supplementary Data [file supp_erv388_Wang_Supplementary_Data_Rev.pdf]

New Isoforms and Assembly of Glutamine Synthetase in the Leaf of Wheat (*Triticum aestivum L.*)

Xiaochun Wang[123], Yihao Wei[3], Lanxin Shi[4], Xinming Ma[1] and Steven M. Theg[4]

Supplementary data

Supplemental Table 1 Identification of the composition of GS_{I}, GS_{II}, and GS_{III} by MS analysis.

Supplemental Fig. S1. GS isoforms in the leaf of different wheat cultivars. Samples were taken at 15 days post emergence. Leaf proteins were separate using native-PAGE with a transferase assay. Lane 1-22 stands for wheat cultivar Luo 3429, Yumai 49, Xinong 509, Aikang 58, Zoumai 27, Zhengmai 366, Zoumai 28, Kaimai 20, Aizao 8, Hengguan 35, Xinmai19, Luomai 24, Zhengmai 101, Xinmai23, Zhongyu 9398, Zhengmai 379, Zhongyuan 6, Aikang58, Zoumai 26, Zhengmai583, Xinong 979, and Yunong 202 respectively.

Supplemental Fig. S2. GS isoforms in wheat chloroplasts (Cp), leaves (L) and roots (R). Soluble proteins were separated using the BNE protocol described, and GS isoforms were detected with a transferase activity assay. The gel shows two replicates of each sample.

Supplemental Fig. S3. GS isoforms as a function of leaf development in wheat. GS isoforms were monitored using native-PAGE (5%) in the first leaf in seedlings of wheat cultivars Yumai 34, 49 and 50 growing with different nitrogen sources (Table 2). Samples were taken at 3(a), 5 (b), 8(c), 11(d), 15(e), 20(f) days post emergence.

Supplemental Fig. S4. GS isoforms in wheat chloroplast, leaf and roots. The soluble proteins from chloroplast (Cp), leaf (L) and roots (R) were separated using the modified BNE protocol described, and GS isoforms were detected with a transferase activity assay.

Supplemental Fig. S5. GS isoforms in wheat leaf (Wl) and roots (Wr), and maize leaf (Ml) and roots (Mr). The soluble proteins from leaves and roots were separated using the modified BNE protocol described and GS isoforms were detected with a transferase activity assay.

Supplemental Fig. S6. Amino acid modifications sites in GS_{II} and GS_{III}.

Supplemental Table S1

Supplemental Table S1 Identification of the composition of GS_{I}, GS_{II}, and GS_{III} in wheat leaf by MS analysis.

GS activity	GS genes coding for
GS_{I}	GS1a, GS1b, GS1c, GSr1, GSr2
GS_{II}	GS2a, GS2b, GS2c
GS_{III}	GS2a, GS2b, GS2c

Fig. S1. GS isoforms in the leaf of different wheat cultivars. Leaf proteins were separate using native-PAGE with a transferase assay. Lane 1-22 stands for wheat cultivar Luo 3429, Yumai 49, Xinong 509, Aikang 58, Zoumai 27, Zhengmai 366, Zoumai 28, Kaimai 20, Aizao 8, Hengguan 35, Xinmai19, Luomai 24, Zhengmai 101, Xinmai23, Zhongyu 9398, Zhengmai 379, Zhongyuan 6, Aikang58, Zoumai 26, Zhengmai583, Xinong 979, and Yunong 202 respectively.

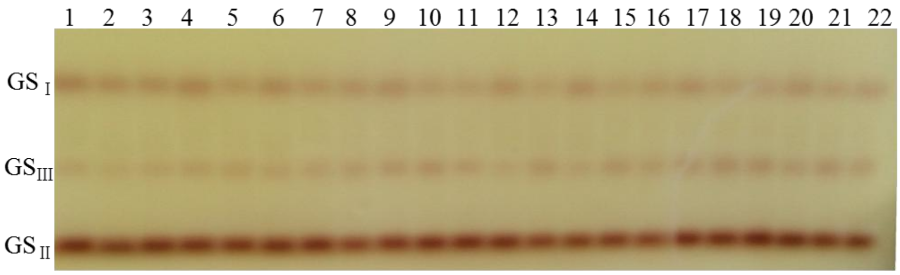

Fig. S2. GS isoforms in wheat chloroplasts (Cp), leaves (L) and roots (R). Soluble proteins were separated using the BNE protocol described, and GS isoforms were detected with a transferase activity assay. The gel shows two replicates of each sample.

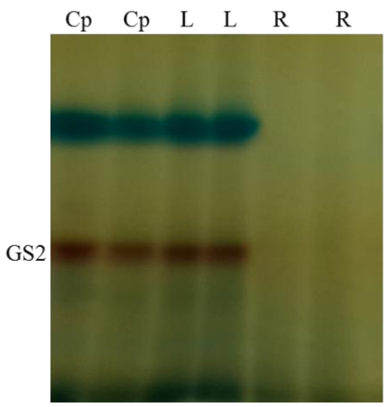

Fig. S3. GS isoforms as a function of leaf development in wheat. GS isoforms were monitored using native-PAGE (5%) in the first leaf in seedlings of wheat cultivars Yumai 34, 49 and 50 growing with different nitrogen sources. Samples were taken 3(a), 5 (b), 8(c), 11(d), 15(e), 20(f) days post emergence.

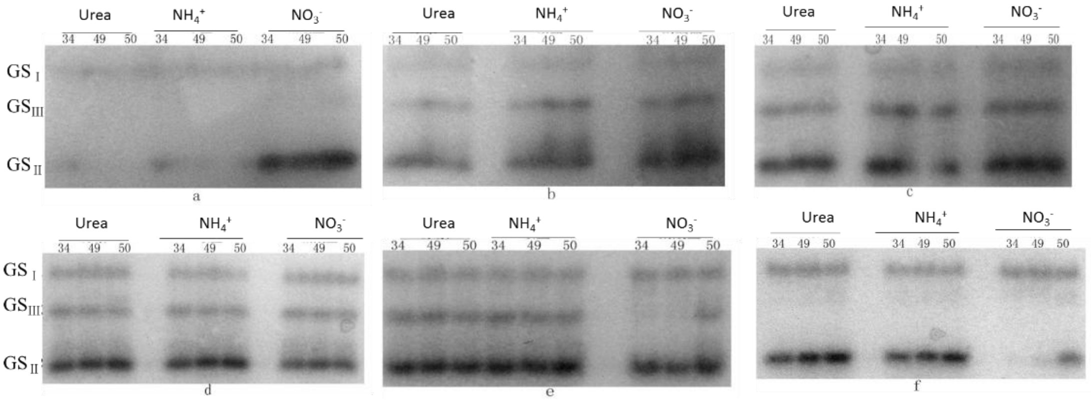

Fig. S4. GS isoforms in wheat chloroplast, leaf and roots. The soluble proteins from chloroplast (Cp), leaf (L) and roots (R) were separated using the modified BNE protocol described, and GS isoforms were detected with a transferase activity assay.

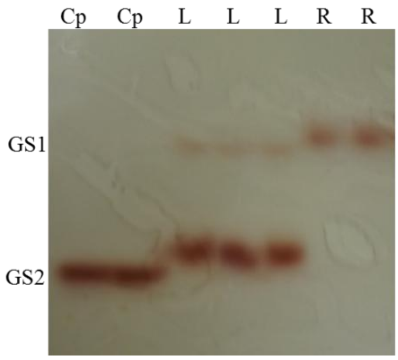

Fig. S5. GS isoforms in wheat leaf (Wl) and roots (Wr), and maize leaf (Ml) and roots (Mr). The soluble proteins from leaves and roots were separated using the modified BNE protocol described and GS isoforms were detected with a transferase activity assay.

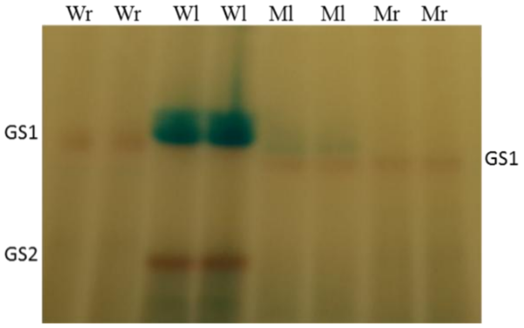

Fig. S6. Amino acid modifications sites in GS$_{II}$ and GS$_{III}$.

A Modification sites in GS$_{II}$

```
1    VLALGPETTG VIQRMQQLLD MDTTPFTDKI IAEYIWVGGS GIDLRSKSRT
51   ISKPVEDPSE LPKWNYDGSS TGQAPGEDSE VILYPQAIFK DPFRGGNNIL
101  VICDTYTPQG EPIPTNKRHM AAQIFSDPKV TAQVPWFGIE QEYTLMQRDV
151  NWPLGWPVGG YPGPQGPYYC AVGSDKSFGR DISDAHYKAC LYAGIEISGT
201  NGEVMPGQWE YQVGPSVGID AGDHIWASRY ILERITEQAG VVLTLDPKPI
251  QGDWNGAGCH TNYSTLSMRE DGGFDVIKKA ILNLSLRHDL HIAAYGEGNE
301  RRLTGLHETA SISDFSWGVA NRGCSIRVGR ETEAKGKGYL EDRRPASNMD
351  PYTVTALLAE TTILWEPTLE AEALAAKKLA LKV
```

B Modification sites in GS$_{III}$

```
1    VLALGPETTG VIQRMQQLLD MDTTPFTDKI IAEYIWVGGS GIDLRSKSRT
51   ISKPVEDPSE LPKWNYDGSS TGQAPGEDSE VILYPQAIFK DPFRGGNNIL
101  VICDTYTPQG EPIPTNKRHM AAQIFSDPKV TAQVPWFGIE QEYTLMQRDV
151  NWPLGWPVGG YPGPQGPYYC AVGSDKSFGR DISDAHYKAC LYAGIEISGT
201  NGEVMPGQWE YQVGPSVGID AGDHIWASRY ILERITEQAG VVLTLDPKPI
251  QGDWNGAGCH TNYSTLSMRE DGGFDVIKKA ILNLSLRHDL HIAAYGEGNE
301  RRLTGLHETA SISDFSWGVA NRGCSIRVGR ETEAKGKGYL EDRRPASNMD
351  PYTVTALLAE TTILWEPTLE AEALAAKKLA LKV
```

Acetyl Dioxidation Oxidation Deamidation
